# Femtosecond Laser Implantation of a 355-Degree Intrastromal Corneal Ring Segment in Keratoconus: A Three-Year Follow-Up

**DOI:** 10.1155/2019/6783181

**Published:** 2019-10-09

**Authors:** Marwa Mahmoud Abdellah, Hatem Gamal Ammar

**Affiliations:** Ophthalmology Department, Sohag University, Sohag, Egypt

## Abstract

**Purpose:**

To evaluate the outcomes of femtosecond laser-assisted implantation of a 355-degree intracorneal ring (ICR) (Keraring) in patients with keratoconus in the three-year follow-up.

**Setting:**

Future Femtolaser Center, Sohag, Egypt.

**Design:**

Prospective interventional case series.

**Patients and Methods:**

A prospective case series of 38 eyes of 26 patients with keratoconus had implantation of the 355-degree ICR keraring after tunnel creation with a femtosecond laser. The uncorrected visual acuities (UCVA) and best-corrected visual acuities (BCVA), sphere, cylinder, and manifest refraction spherical equivalent (SE), and mean keratometry (*K*), *K* max, and *K* min were evaluated preoperatively and 3, 6, 12, 24, and 36 months postoperatively, and all complications were reported.

**Results:**

38 eyes of 26 patients with mean age 25.92 ± 5.44 years were enrolled in the study, 11 were males (**42.3%**). The mean UCVA improved from 0.93 ± 0.21 to 0.63 ± 0.21 logMAR (*P* ≤ 0.001) and the mean BCVA from 0.67 ± 0.22 to 0.43 ± 0.26 logMAR (*P* < 0.001). The mean sphere, cylinder, and spherical equivalent have been changed dramatically from preoperative to 3 month postoperative, which is statistically significant (*P* ≤ 0.001), and the changes between 1 and 2 years and 2 and 3 years are also considerable and statistically significant; the *K* max and *K* min and *K mean* improved and the changes were statistically significant (*P* ≤ 0.001), and the changes between one, two, and three years were also statistically significant. The safety and efficacy indices were changed through the three-year follow-up. The complications were corneal neovascularization (36.84%), corneal melting (26.3%), and ring extrusion (31.5%) at the end of the study.

**Conclusions:**

Implantation of a 355-degree intracorneal keraring using femtosecond laser improved the visual, refractive, and topographic parameters in keratoconus patients, with a high rate of ICR extrusion and instability. The study has been registered for the Pan African Clinical Trial Registry (http://www.pactr.org) database within No: PACTR201810796878908 on 29 October 2018.

## 1. Introduction

Keratoconus is a disease characterized by stromal weakening followed by corneal protrusion leading to irregular astigmatism, which impairs the vision a lot, and its exact etiology cannot be determined; actually, many factors are accused for keratoconus occurrence [1].

In previous studies, the prevalence of keratoconus in different countries ranged from 0.3 per 100,000 in Russia to 23 per 1000 in central India (0.0003%–2.3%) [2, 3], and in recent studies about incidence in upper Egypt, the prevalence was as high as17.5% among the populations who are seeking refractive surgery [4].

So, many techniques are introduced as therapeutic choices for the management keratoconus to give more stability and better optical improvement. The procedures are different, ranging from crosslinking, intracorneal ring segment, and lamellar keratoplasty.

ICRS, small synthetic devices, are designed to be implanted within the corneal stroma aiming to induce a change in the geometry and refractive power of the tissue [5]. The first idea of corneal rings was introduced by Blevatskaya in 1966 [6]. Twenty years ago, the design of ring segments was introduced to correct the myopic refractive error [5]. Then, the rings were shown that they were effective in keratoconus management.

Many types are introduced in the market, and the main types are Keraring (Mediphacos) and Intacs (Addition technologies). In recent years, kerarings are polymethyl methacrylate (PMMA) characterized by triangular cross-sectional design and ultraviolet blocking effect, and they are available in variable thickness and arc length. Mediphacos developed a new *n* interrupted ring of 355°, which is available in a diameter of 5.7 mm and a thickness ranging from 200 to 300 *μ*m [5], designed mainly for nipple keratoconus [5].

There are few studies published in the literature reporting the success of these rings in visual and refractive improvement in patients with central keratoconus [7, 8]; however, long follow-up studies to show its safety and efficacy within years are not available.

The normal orthogonal arrangement of the stromal fibers is distorted in the keratoconus patients so the course of intrastromal rings within these fibers is unpredictable [9]; hence further follow-up studies are needed especially as safety and efficacy are changed within time.

## 2. Materials and Methods

This prospective, consecutive, interventional study included 38 eyes from 26 patients with central keratoconus (15 females and 11 males), with a mean age of 25.92 ± 5.44 years (range 18–38 years).

The study was approved by the Ethical Committee of Faculty of Medicine, Sohag University, and followed the tenets of the Declaration of Helsinki. After full explanation about the purpose and procedures of the study, an informed written consent was obtained from the patients. The inclusion criteria were age greater than 20 years, central type keratoconus, which is diagnosed by equal or more than 50% of the cone is within the 3.0 mm zone on the posterior elevation map of the Pentacam rotating Scheimpflug device (OCULUS Optikgerate GmbH, Wetzlar, Germany) [10], moderate keratoconus and severe keratoconus cases according to the steepest keratometric reading [11], clear central cornea, pachymetry of above 400 microns, and no visual dysfunctions other than keratoconus. Contact lens wear was discontinued two weeks prior to the examination. Exclusion criteria were active allergic conjunctivitis, a history of keratorefractive surgery on the operative eye, dry eye, pregnancy, lactation, corneal stromal disorders, corneal erosion syndrome, corneal scars, previous herpes keratitis, autoimmune or immunodeficiency diseases, eccentric cone, presence of cataract, and glaucoma or retinal diseases.

A full detailed ophthalmic examination was performed preoperatively and postoperatively, including uncorrected visual acuity (UCVA), best spectacle-corrected visual acuity (BSCVA), manifest refraction, spherical equivalent (SE), keratometry (*K*) readings (max *K*, min *K*, and mean *K*), and ultrasound pachymetry. Anterior and posterior corneal surface topography were examined by Scheimpflug imaging using Pentacam; visual acuity was measured using decimal values and then converted to logMAR for statistical analysis.

The safety of implantation of Keraring 355° in patients with keratoconus was assessed using a refractive surgery safety index (safety index = postoperative best-corrected visual acuity ÷ preoperative best-corrected visual acuity in decimal values) [12].

Efficacy was calculated using a refractive surgery efficacy index, which is the mean postoperative UCVA/mean preoperative BCVA [12].

### 2.1. Surgical Technique

The procedure was performed under topical anesthesia using benoxinate hydrochloride followed by sterilization using Betadine (5% povidine iodine), and a sterile plastic sterile was applied to draw away the lashes, and then the speculum was used to open the eye.

**Figure 1:**
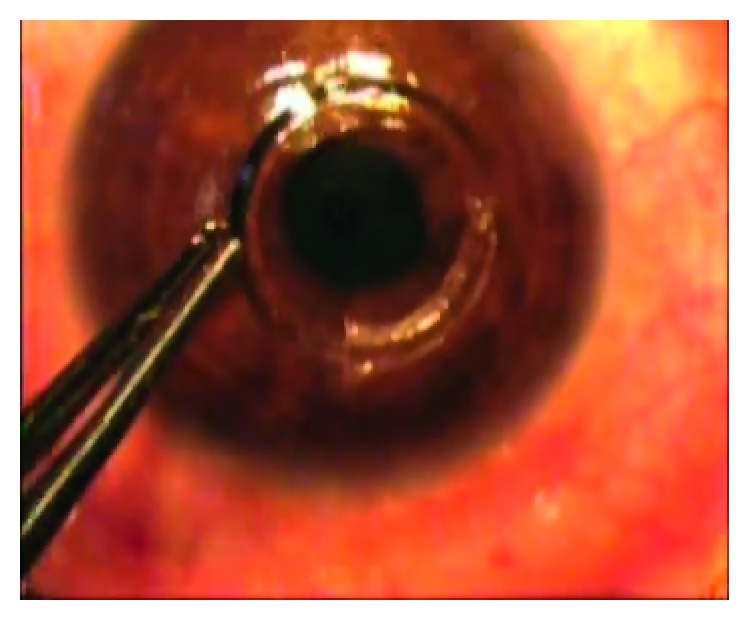
The insertion of the 355-degree keraring in the cornea after femtosecond tunnel formation.

Procedures started by marking the corneal center when the patient was fixating at the fixating light, then followed by application of the suction ring onto the cornea with great care of corneal centralization within the suction ring. The corneal tunnel was created using the femtosecond laser (*iFS Advanced Femtosecond Laser*, *Abbott*, *USA*), with a power of 5 mJ. Passing a spatula was performed through the limbs of the tunnel to check its patency. The 355-degree Keraring (Mediphacos Inc., Belo Horizonte, Brazil) segments were implanted carefully (Figure 1).

The tunnel depth was set at 300 microns subsequently; a silicone-hydrogel bandage contact lens (Bausch and Lomb) was placed on the cornea. Postoperatively, patients were given combined dexamethasone and tobramycin drops (TobraDex; Alcon Laboratories, Novartis, Inc., Fort Worth, Texas, USA) 4 times a day, moxifloxacin 0.5% drops (Vigamox, Alcon Laboratories, Novartis, Inc., Fort Worth, Texas, USA) 6 times a day, and nonpreserved artificial tears (Systane, Alcon Laboratories, Inc., Fort Worth, Texas, USA) 5 times a day. Moxifloxacin drops was discontinued one week postoperatively and dexamethasone drops tapered off during 3 weeks. Bandage contact lenses were removed one day postoperative. Patients were scheduled for postoperative clinical examination at 1st day and after 1 month, 3 months, 6 months, 12 months, 24 months, and 36 months. Intraoperative and postoperative complications were recorded. All the cases were operated by the same surgeon.

### 2.2. Statistical Analysis

Data were analyzed using SPSS computer program version 22.0.

Quantitative data were expressed as means ± standard deviation, median, and range. Qualitative data were expressed as number and percentage. The data were tested for normality using the Shapiro–Wilk test. The nonparametric Wilcoxon signed-rank test was used because the data were not normally distributed. A 5% level was chosen as a level of significance in all statistical tests used in the study.

### 2.3. Results

**Figure 2:**
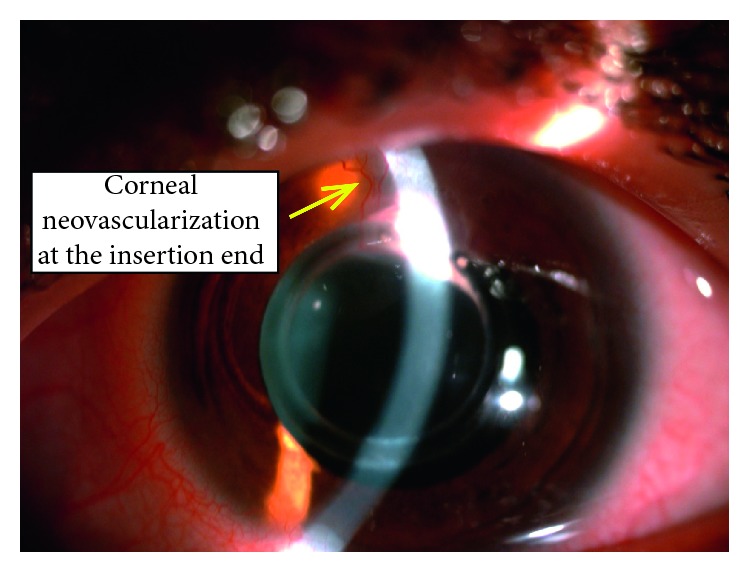
Neovascularization of the cornea at the insertion end of the 355-degree ring.

**Figure 3:**
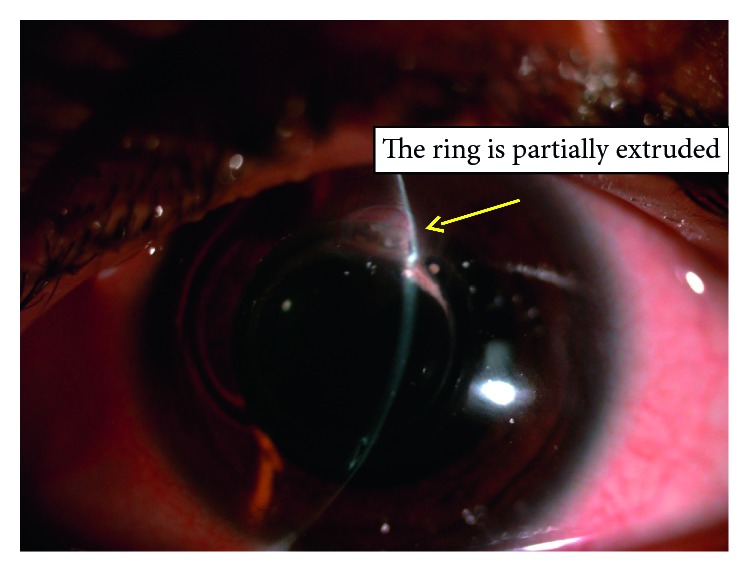
The ring partially extruded.

**Figure 4:**
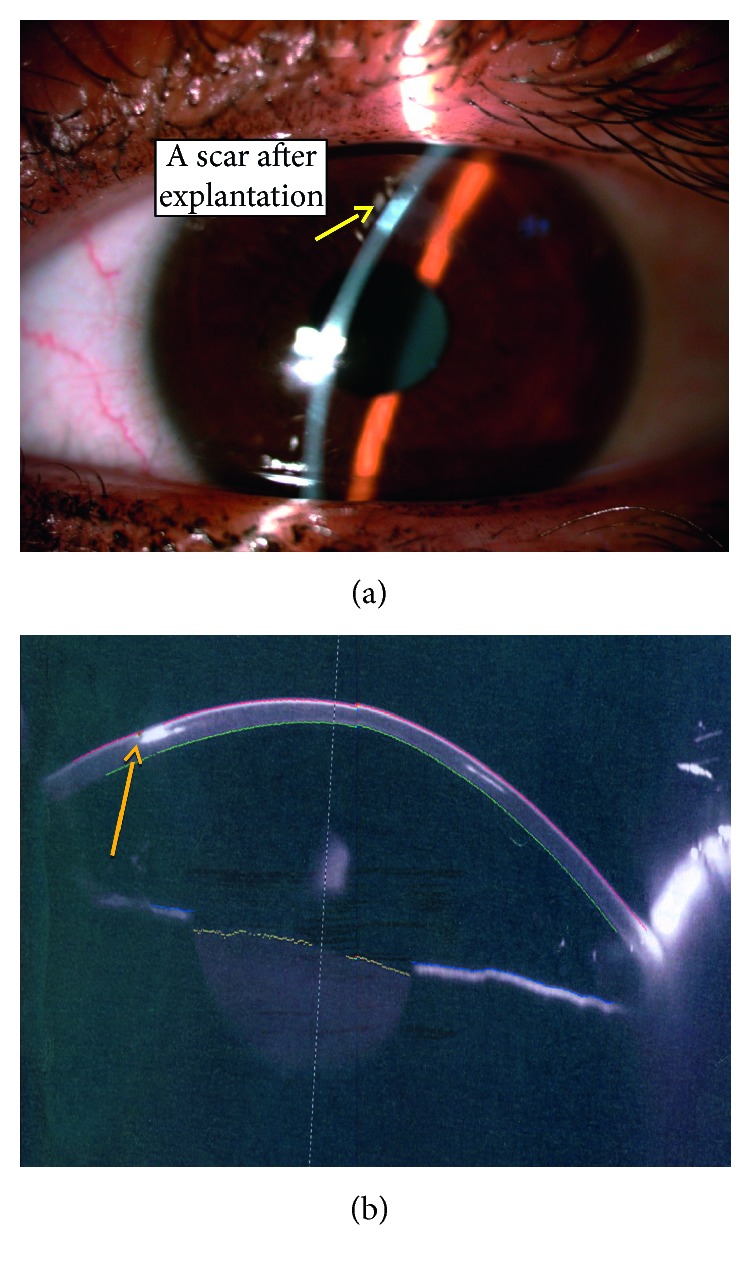
(a) Slit lamp photo of the cornea showing a scar after ring explantation and (b) Scheimpflug imaging of the cornea showing the scar.

Thirty eight eyes of twenty six patients were enrolled in the study; sixteen eyes (42.1%) were diagnosed as moderate keratoconus with *K* max ≤ 52 D, while twenty-two eyes (57.9%) were diagnosed as severe keratoconus with *K* max >52 D using the steepest *K* reading. The mean age was 25.92 ± 5.44 years.  Table 1 shows the patient demographics. The mean UDVA improved from 0.93 ± 0.21 logMAR to 0.63 ± 0.21 (*P* ≤ 0.001), and after 36 months, the mean BCVA from 0.67 ± 0.22 logMAR to 0.43 ± 0.26 logMAR (*P* < 0.001), as shown in Table 2. The mean sphere, cylinder, and spherical equivalent have been changed dramatically from preoperative to 3 months postoperative, which are statistically significant, and the changes between 1 and 2 years and 2 and 3 years were also considered due to the high rate of ring extrusion, shown in detail in Table 3; the *K* max and *K* min and *K* mean changed a lot, and the changes between preoperative and postoperative were statistically significant (*P* ≤ 0.001); the changes between one, two, and three years were statistically significant, Table 4. The safety and efficacy indices were changed through the three-year follow-up (Table 5). The complications, which were reported, ranged from corneal neovascularization (Figure 2), corneal melting, and ring extrusion (Figure 3), which ended by ring explantation (Figure 4) in about the third of cases at the end of the three-year follow-up, Table 6.

## 3. Discussion

The aim of ICRS surgery is to induce a geometric change in the central corneal curvature, thus, reducing the refractive error and the mean keratometry and improving the visual acuity. Additionally, corneal remodeling results in an improvement in the optical quality of the cornea, and a reduction in optical aberrations can also be expected [13–15]. So, the aim of this study was to report the efficacy and safety of femtosecond laser implantation of 355-° intrastromal corneal rings in the management of keratoconus; actually, few reports discussed the efficacy and safety of this procedure within a short time of follow-up.

According to the results obtained from this study, the femtosecond laser implantation of a 355-degree intrastromal corneal ring segment was successful according to the definition of success defined by Jorge [15], who defined successful surgery as one of the following characteristics at six months postoperatively: either an improvement in one or more lines of uncorrected visual acuity (UCVA) or BCVA or a decrease in two or more D of spherical equivalent. “Stable cases” were defined as cases without significant changes in corneal topography (<1 D in the mean keratometry mean) over 12 months preoperatively [15].

The ICRS surgery acts by regularizing the anterior corneal surface, thus, decreasing myopia and regular and irregular astigmatism. The introduction of the femtosecond laser in this procedure gave more advantages such as more safety, more accuracy, and easily accessible by the surgeon [16–18], which is advantageous over the study performed by Jadidi et al. [7].

Regarding the results, the UCVA changed dramatically after the procedure, which is statistically significant. This agreed with Jadidi study who implanted 355-degree kerarings via a mechanical technique using keratome: the UCVA improved from preoperative to postoperative at 1 and 3 months and the change between 3 and 6 months showed an improvement which was statistically significant [7], while in this study the change between the 12 and 24 months and between 24 months and 36 months were worse and statistically significant, which is explained by the high rate of ICRS extrusion which affects the final UCVA mean.

The *K* readings including the *K* max, *K* min, and *K* mean changed a lot postoperative and the change was statistically significant (*P* ≤ 0.001), which implied the ability of the rings to change the convexity and geometry of the cornea; however, the mean K readings were increased in the subsequent follow-ups due to high incidence of the ring extrusion followed keratoconus progression after ring explantation.

We observed statistically significant reductions in myopia and cylinder. The changes were of a large magnitude, with a mean change in the sphere of 3.36 D and a mean change in the refractive cylinder of 2.01 D after the first year (*P* = 0.001). These levels of refractive change were consistent with those previously reported after MyoRing implantation with mechanical dissection [18–20] and with Femtolaser-assisted implantation of MyoRing implantation carried out by Saeed [21]; in this study, there was an increase in the myopia and the astigmatism values, which was statistically significant (*P* = 0.004 and *P* = 0.003, respectively); between 12-month follow-up and 24-month follow-up correlated to the increased number of cases complicated by ring extrusion, follow-up studies regarding the MyoRing implantation are opposite.

Regarding the complications of this type of rings, it was found there was a high rate of corneal melting at the site of insertion and ring extrusion; most of corneal melting cases began after 6 months postoperative; in this study, there was a high incidence of six ring extrusion cases (15.78%) after 12 months and twelve cases (31.5%) at 36 months; it was observed that the cases of corneal melting and ring extrusion preceded by a corneal neovascularization at the site of the ring insertion, which may imply a state of silent inflammation with these rings; the ring extrusion is previously mentioned by Jadidi in a small cases series using this 355-kerarings [8]. This high rate of corneal neovascularization and corneal melting which ended by ring explantation was not mentioned in this high percentage before; corneal neovascularization was 0.2% in a previous study of 850 eyes [22]. This claims a lot about the design of these rings. In our explanation; the circular design of the ring is to be about a complete circle which inserted through a tight tunnel, making the ring perform a persistent pressure on the upper corneal stromal fibers causing its melting; otherwise, in two separate segments design, there is a space to relieve pressure transmitted to the stromal fibers through the segment-free tissues. It is worth to mention this high rate of ring extrusion was quite the same in both moderate and severe stages of keratoconus cases; so the high rate of ring extrusion mostly related to the design of rings rather than the stage of keratoconus Although the MyoRing, which is a circular complete 360-degree ring, but it is inserted through a pocket, not a tunnel, making the pressure transmitted to a large area of stroma with less effect on stromal fibers. This agreed with previous results of long-term studies regarding the MyoRing as no cases of ring extrusion were reported. [8, 21–23]. However, comparing with a new incomplete 320 intracorneal stomal ring, it was found that the reduction in myopia and astigmatism values and corneal flattening effect are comparable with the 355 rings in this study; however, no cases of ring extrusion were reported in six-month follow-up [24], but there were few reported cases of ring migration [25]; other models of keraring such as 210 ring showed a success in improving the BSCVA; no cases of ring extrusion were reported with them [26]. It was supposed earlier that long-term stability of ICRS implantation depended on the progression pattern of keratoconus at the time of surgery. Thus, stable keratoconus with less progression gave more stable results and more success [27], and Alio suggested that insertion of intrastromal rings should be performed after confirmation of stability in keratoconus patients [28], but this cannot be applied in all cases.

To our knowledge, it is first study which studied the three-year follow-up of this model of ICSR (355-keraring), which highlights the high rate of ring extrusion accompanied with this ring model, agreeing with few previous reports. However, some limitation with this study which should be included in further studies is higher order aberrations analysis, to be compared with other type of ICSR; further studies are needed to study the effect of corneal cross-linking as a compound procedure with 355-keraring to improve its stability and efficacy.

## 4. Conclusion

Implantation of a 355-degree intracorneal keraring using femtosecond laser improved the visual, refractive, and topographic parameters in keratoconus patients, with a high rate of ICR extrusion and instability in the three-year follow-up.

## Figures and Tables

**Table 1:** Patient demographics.

Number of patients	26
Age	
Mean ± SD	25.92 ± 5.44
Median (range)	24.5 (18–38)
Gender	
Male, n (%)	11 (42.3)
Female, n (%)	15 (57.69)

**Table 2:** Comparison between preoperative and postoperative UCVA and BCVA measures.

	Preoperative	3 months postoperative	6 months postoperative	12 months postoperative	24 months postoperative	36 months postoperative	*P* value	*P*1	*P*2
UCVA (logMAR)									
Mean ± S.D.	0.93 ± 0.21	0.46 ± 0.18	0.47 ± 0.18	0.49 ± 0.18	0.54 ± 0.18	0.63 ± 0.21	<0.001	<0.001	0.001
Median (range)	0.9 (0.5–1.3)	0.4 (0.2–0.8)	0.45 (0.2–0.8)	0.45 (0.2–0.8)	0.6 (0.2–0.8)	0.6 (0.3–1.1)			
BCVA (logMAR)									
Mean ± S.D.	0.67 ± 0.22	0.29 ± 0.16	0.29 ± 0.15	0.37 ± 0.23	0.39 ± 0.24	0.43 ± 0.26	<0.001	0.248	0.052
Median (range)	0.6 (0.3–1.1)	0.25 (0.1–0.8)	0.3 (0.1–0.8)	0.3 (0.1–0.9)	0.3 (0.1–0.9)	0.3 (0.1–1)			

*P* value compared the six repeated measures and was calculated by Friedman's two-way ANOVA test. *P*1 compared 12 months postoperative and 24 months postoperative measures and was calculated by using Wilcoxon signed-rank test. *P*1 compared 24 months postoperative and 36 months postoperative measures and was calculated by Wilcoxon signed-rank test. *P* value <0.05 is statistically significant Notes: UCVA: uncorrected visual acuity; BCVA: best-corrected visual acuity; D: diopters; logMAR, logarithm of the minimum angle of resolution; SD: standard deviation.

**Table 3:** Comparison between preoperative and postoperative sphere, cylinder, and spherical equivalent (SE) measurements.

	Preoperative	12 months postoperative	24 months postoperative	36 months postoperative	*P* value	*P*1	*P*2
Sphere							
Mean ± S.D.	−9.68 ± 3.08	−6.32 ± 2.37	−7.13 ± 3.09	−7.45 ± 3.2	<0.001	0.004	0.129
Median (range)	−8.75 (−4.25–−16)	−5.75 (−3.5–−12.5)	−7.25 (−3.5–15)	−7.75 (−3.75–−15.5)			
Cylinder							
Mean ± S.D.	−5.82 ± 1.55	−3.81 ± 0.81	−4.21 ± 1.24	−4.32 ± 1.24	<0.001	0.003	0.198^*∗*^
Median (range)	−6.25 (−3.25–−8.5)	−3.88 (−2–5.5)	−4 (−2–7)	−4 (−2–6.75)			
Spherical equivalent SE							
Mean ± S.D.	−12.55 ± 3.64	−8.25 ± 2.62	−8.72 ± 3.39	−9.62 ± 3.73	<0.001	0.173	0.009
Median (range)	−12 (−6.12–−19.75)	−7.75 (5–−14.75)	−7.75 (−5–−19.5)	−9.94 (−5–−18.75)			

*P* value compared the six repeated measures and was calculated by Friedman's two-way ANOVA test. *P*1 compared 12 months postoperative and 24 months postoperative measures and was calculated by Wilcoxon signed-rank test. *P*1 compared 24 months postoperative and 36 months postoperative measures and was calculated by Wilcoxon signed-rank test. ^*∗*^*P* value was calculated by paired samples *t* test. *P* value <0.05 is statistically significant. SE: spherical equivalent.

**Table 4:** Comparison between preoperative and postoperative *K* max, *K* min, and *K* mean measures.

	Preoperative	3 months postoperative	6 months postoperative	12 months postoperative	24 months postoperative	36 months postoperative	*P* value	*P*1	*P*2
*K* max									
Mean ± SD	53.82 ± 4.1	47.48 ± 2.16	47.44 ± 2.11	48.04 ± 2.39	49.51 ± 3.73	50.47 ± 4.08	<0.001	<0.001	0.001
Median (range)	54.6 (47.6–61.2)	48 (43–52)	47.3 (44–51.3)	47.75 (44.2–56.1)	48.95 (44.1–61.5)	49.5 (44.5–62)			
*K* min									
Mean ± SD	48.83 ± 2.84	45.23 ± 1.99	45.41 ± 1.82	45.8 ± 2.13	46.53 ± 2.46	47.01 ± 2.4	<0.001	<0.001	0.001
Median (range)	49.5 (45–53.2)	45.05 (42–48)	45.75 (41.9–48)	45.6 (42–50)	46 (42.7–55)	46 (43–55)			
*K* mean									
Mean ± SD	51.29 ± 3.49	46.29 ± 1.48	46.17 ± 1.67	46.57 ± 1.62	47.12 ± 1.62	47.51 ± 1.95	<0.001	0.001	0.055^*∗*^
Median (range)	52.95 (46–56)	46.6 (43.8–48.4)	46.6 (43–48)	46.55 (43.8–49)	47.35 (44–50)	47.6 (44–52)			

*P* value compared the six repeated measures and was calculated by Friedman's two−way ANOVA test.*P*1 compared 12 months postoperative and 24 months postoperative measures and was calculated by the Wilcoxon signed-rank test.*P*1 compared 24 months postoperative and 36 months postoperative measures.

**Table 5:** The efficacy index and safety index of the procedure.

	3 months	6 months	12 months	24 months	36 months
Safety index	2.27	2.27	1.95	1.81	1.70
Efficacy index	1.59	1.59	1.45	1.32	1.09

**Table 6:** The postoperative complications.

	12 months			24 months			36 months		
	Total no.	Moderate keratoconus	Severe keratoconus	Total no.	Moderate keratoconus	Severe keratoconus	Total no.	Moderate keratoconus	Severe keratoconus
Infectious keratitis	0	0	0	0	0	0	0	0	0
Corneal neovascularization	7 (18.4%)	3 (18.75%)	4 (18.18%)	11 (28.9%)	5 (31.16%)	6 (27.27%)	14 (36.84%)	6 (37.5%)	8 (36.36%)
Corneal melting	5 (13.15%)	2 (12.5%)	3 (13.63%)	8 (21%)	3 (18.75%)	7 (22.73%)	10 (26.3%)	4 (25%)	6 (27.27%)
Ring extrusion	6 (15.78%)	2 (12.5%)	4 (18.18%)	10 (26.3%)	4 (25%)	6 (27.27%)	12 (31.5%)	5 (31.25%)	7 (31.82%)

## Data Availability

Data are available on request (videos, Pentcams, and photos) to the corresponding author.

## Supplementary Materials

Supplementary Materials An edited video shows femtosecond tunnel creation and 355-degree keraring insertion.
